# Extra-pancreatic manifestations reported in association with pancreatitis; an international survey report

**DOI:** 10.1371/journal.pone.0288337

**Published:** 2023-07-11

**Authors:** Chinenye R. Dike, Qin (Kiki) Sun, Lola Rahib, Megan Golden, Maisam Abu-El-Haija

**Affiliations:** 1 Department of Pediatrics, Division of Pediatric Gastroenterology, Hepatology and Nutrition, University of Nebraska Medical Center and Children’s Hospital & Medical Center, Omaha, NE, United States of America; 2 Department of Pediatrics, Division of Pediatric Gastroenterology, Hepatology and Nutrition, University of Alabama at Birmingham, Birmingham, AL, United States of America; 3 Department of Pediatrics, Division of Biostatistics and Epidemiology, Cincinnati Children’s Hospital Medical Center, Cincinnati, OH, United States of America; 4 Mission: Cure, New York, New York, United States of America; 5 Department of Pediatrics, Division of Pediatric Gastroenterology, Hepatology and Nutrition, Cincinnati Children’s Hospital Medical Center, Cincinnati, OH, United States of America; 6 University of Cincinnati, Department of Pediatrics, Division of Pediatric Gastroenterology, Hepatology and Nutrition, Cincinnati, OH, United States of America; Hacettepe University: Hacettepe Universitesi, TURKEY

## Abstract

**Background/objectives:**

Local and systemic manifestations have been reported in association with pancreatitis, anecdotally. However, a systematic collection on the prevalence of each of these symptoms in pancreatitis is lacking. We aimed to determine the prevalence of symptoms and diagnoses reported by a cohort of patients with pancreatitis, refer to as “extra pancreatic manifestation of pancreatitis”.

**Methods:**

Cross-sectional study approved by the IRB and administered through a REDCap survey by “Mission: Cure”, a nonprofit organization.

**Results:**

Of the 225 respondents analyzed; 89% were adults, 69% females, 89% Caucasians with 74% residing in the USA. 42% of children and 50% of adults reported exocrine pancreatic insufficiency while 8% of children and 26% of adults reported DM. Type 3c DM was reported in all children and 45% of adult DM cases. Children were diagnosed with genetic or hereditary pancreatitis more frequently compared to adults (33.3% versus 8%; p = <0.001). Significantly more symptoms and diagnoses were reported by adults when compared to children including nighttime sweats, bloating, or cramping, greasy or oily stools, feeling cold and GERD with p values of 0.002, 0.006, 0.046, 0.002 and 0.003 respectively.

**Conclusions:**

Adults with pancreatitis frequently report symptoms not known to be associated with pancreatitis. Studies investigating mechanisms for these associated symptoms should be explored.

## Introduction

Although extra-pancreatic manifestations have long been reported in association with pancreatitis, there are gaps in literature on the prevalence of most of these diagnoses and symptoms associated with pancreatitis. Many of the reported manifestations are limited to case reports, case series, single center studies and few systematic reviews [1–5].

IgG4- related disease including autoimmune pancreatitis has been described since the early 2000s in association with other extra-pancreatic manifestations including but not limited to retroperitoneal fibrosis [2], head and neck lesions (Riedel thyroiditis and thyroid dysfunction [5], inflammation of the orbits including periocular xanthogranulomas, pituitary hypophysitis, orbital lymphoid hyperplasia, salivary gland inflammation [1, 3]), pulmonary lesions including adult onset asthma [4], inflammatory bowel disease [6, 7], vascular lesions such as aortitis, renal dysfunction from tubulointerstitial nephritis [8] with perinephric abscess [9], sclerosing cholangitis [10], hepatosplenomegaly [11] and impaired gastric emptying [12].

Other extra pancreatic manifestations that have been described in association with pancreatitis include panniculitis, polyarthritis (PPP) syndrome [13]. Cutaneous manifestations reported with pancreatitis include acanthosis nigricans, necrolytic migratory erythema, livedo reticularis and hemorrhagic skin lesions seen in severe acute pancreatitis (AP) [14]. Other symptoms that have been described with AP include fatigue [13], abdominal wall abscess and epididymoorchitis [15].

While extra-pancreatic manifestations in autoimmune (Ig4 related) pancreatitis have been described, and there are studies focusing on exocrine and endocrine insufficiency, data on the prevalence of extra pancreatic manifestations in AP and chronic pancreatitis (CP) are limited. Given the gaps in literature on the prevalence of some of these symptoms, the aim of this study is to systematically evaluate and compare the prevalence of extra-pancreatic manifestations reported by adults versus children with pancreatitis through an online questionnaire.

## Methods

This is a cross-sectional anonymous survey approved by the Institutional Review Board at the University of Nebraska Medical Center (UNMC) as an exempt study: 0186-22-EX. The REDCap (Research Electronic Data Capture) administered anonymous survey used in this study was developed through several meetings by study investigators, C.D, M.A.H and personnel from Mission: Cure, including an adult patient with pancreatitis and the caregiver of a child with pancreatitis. The survey was launched via Mission: Cure email thread and social media from April 6^th^ to May 13^th^, 2022. During this period (April 6^th^ to May 13^th^, 2022), patients with pancreatitis and their caregivers who were part of the social support or network with Mission: Cure, were sent weekly reminders to complete the REDCap survey. Data from completed surveys was automatically available in REDCap for research purposes. Available data from completed surveys was assessed by research personnel for data analysis and further research purposes, 3 days after survey had closed to potential participants on May 16^th^, 2022. Neither written nor oral consent was obtained since this was an anonymous survey administered via social media. However, we added a few sentences prior to survey questions indicating that completion of survey was voluntary, completion of survey indicated consent and children were not allowed to complete surveys, but their adult parents or legal guardians should complete survey on their behalf. The survey was administered and analyzed anonymously.

The survey was administered in English and divided into 4 sections: demographic, baseline clinical information, symptoms, and diagnoses. Our primary outcomes were extra pancreatic symptoms reported in adults versus children and our secondary outcomes were comparing known pancreatic symptoms reported in children versus adults.

### Statistical analysis

Descriptive statistics (counts and percentages) were used to describe the cohort and by age groups (<18 years versus ≥18 years). Chi-square test or Fisher’s exact test were used to compare groups for baseline characteristics, symptoms, and diagnoses between the two age groups. A 2-sided p value <0.05 was used to determine the significance of variables in all analyses. Data analyses were performed using SAS version 9.4 (SAS Institute, Cary, North Carolina).

## Results

**Fig 1:**
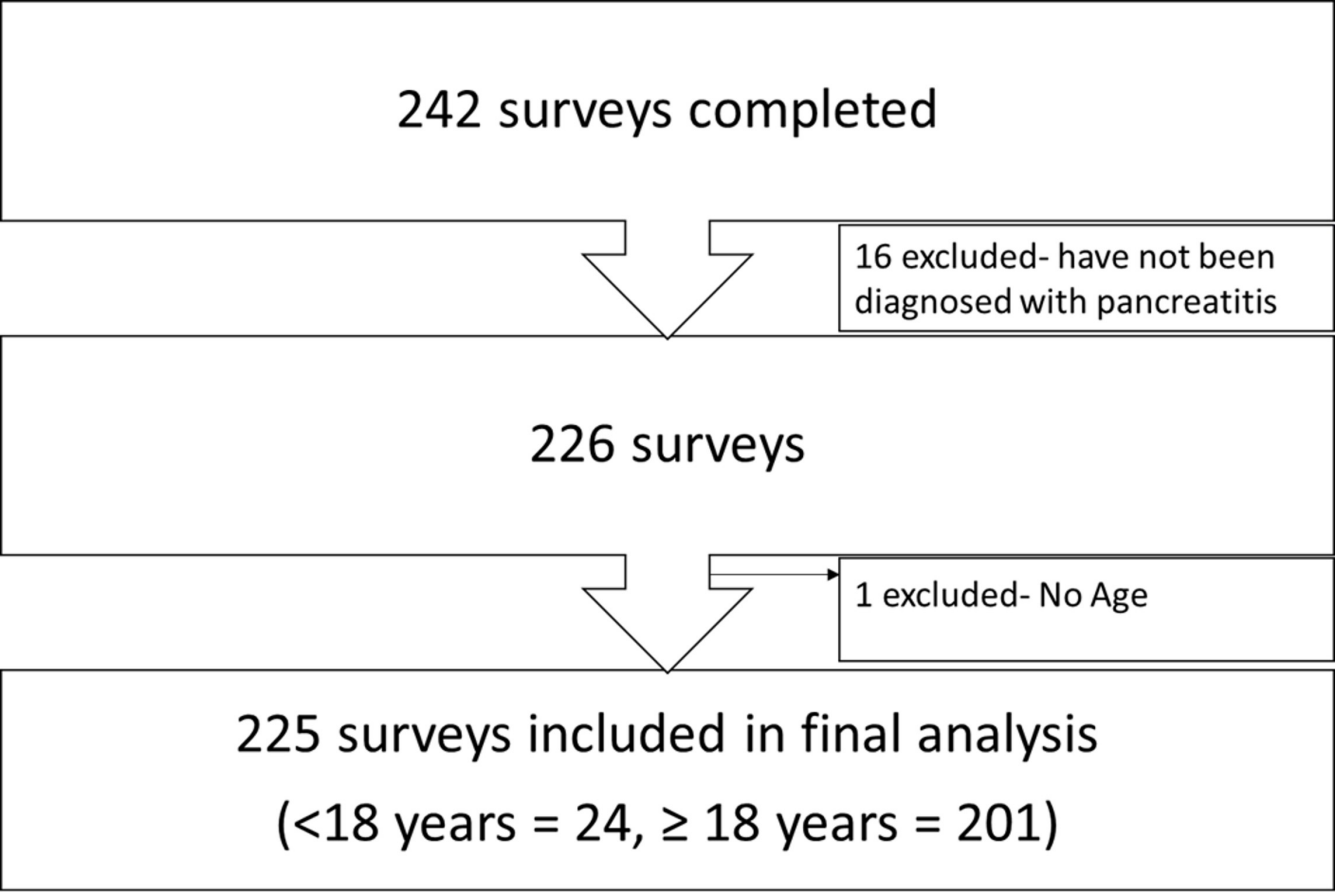
Participant flow chart.

**Fig 2:**
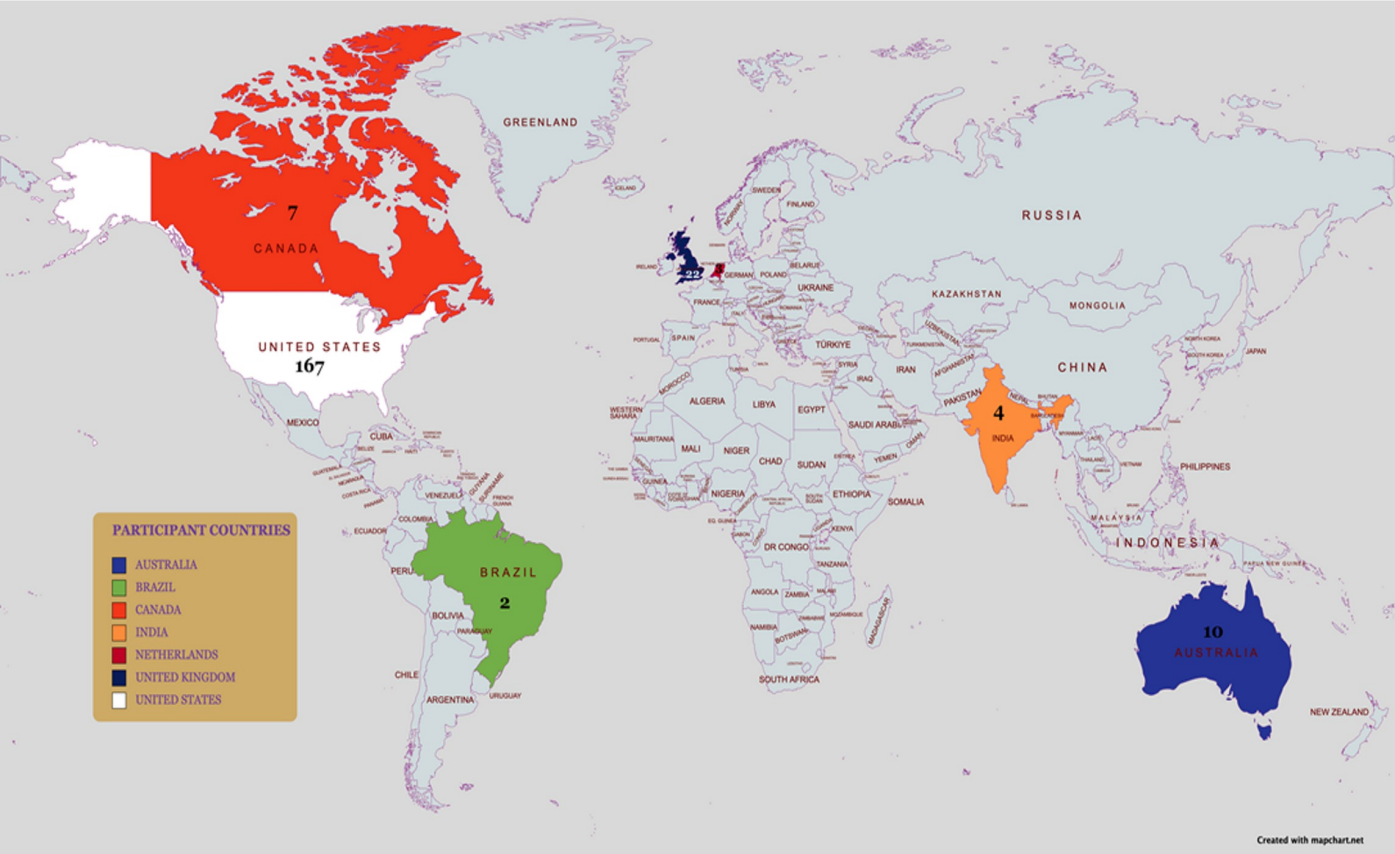
Map of countries with > 1 participant or survey respondent.

A total of 242 respondents completed the survey. Respondents who did not report a diagnosis of pancreatitis by a health provider or age were excluded. 225 surveys were included in the final analysis (Fig 1). Of the 225 respondents included, 201 (∼89%) respondents were 18 years or older (Table 1). Most survey respondents resided in the USA (∼74%) (Fig 2 and Table 1), were females (∼ 69%) and of Caucasian race (∼89%). About 35% of respondents reported having pancreatitis diagnosis for 2–5 years. The most common pancreatitis diagnosis was acute recurrent pancreatitis in the pediatric cohort (∼ 38%) and chronic pancreatitis in the adult cohort (∼67%) (Table 2). Furthermore, more children were diagnosed with hereditary pancreatitis compared to adults (33.3% versus 8%; p < 0.001) and more children reported genetic mutations: PRSS1 mutations (29.2% vs. 6%), SPINK (25% vs. 3%), CFTR mutations (20.9% vs 6.5%) p values of < 0.001, < 0.001 and 0.014 respectively (Table 2).

**Table 1:** Participant demographics.

Age	Overall	<18 years N (column %)	≥18 years N (column %)
**Formal pancreatitis diagnosis**			
Yes	225	24 (10.7)	201 (89.3)
**Gender**			
Female	155	17 (70.8)	138 (68.7)
Male	69	7 (29.2)	62 (30.9)
Gender variant/ non-conforming	1	0 (0)	1 (0.4)
**Race/ ethnicity**			
White	201	17 (70.8)	184 (91.5)
Hispanic, Latino, or Spanish	13	4 (16.7)	9 (4.5)
Black or African American	2	0 (0)	2 (1.0)
Middle Eastern or North African	3	1 (4.2)	2 (1.0)
Asian	7	0 (0)	7 (3.5)
American Indian or Alaska Native	3	0 (0)	3 (1.5)
Native Hawaiian or Other Pacific	0	0 (0)	0 (0)
Islander	0	0 (0)	0 (0)
Prefer not to answer	4	3 (12.5)	1 (0.5)
Other: ………………….			
**Country**			
Australia	10	4 (16.7)	6 (3.0)
Brazil	2	0 (0)	2 (1.0)
Canada	7	1 (4.2)	6 (3.0)
Denmark	1	0 (0)	1 (0.5)
India	4	0 (0)	4 (2.0)
Iceland	1	0 (0)	1 (0.5)
Israel	1	1 (4.2)	0 (0)
Italy	1	0 (0)	1 (0.5)
Kuwait	1	0 (0)	1 (0.5)
Latvia	1	1 (4.2)	0 (0)
Netherlands	3	0 (0)	3 (1.5)
Nicaragua	1	1 (4.2)	0 (0)
Thailand	1	0 (0)	1 (0.5)
UAE	1	0 (0)	1 (0.5)
UK	22	2 (8.3)	20 (10.0)
USA	167	14 (58.3)	153 (76.1)
Trinidad and Tobago	1	0 (0)	1 (0.5)
**Number of years of pancreatitis diagnosis**			
<2 years	36	5 (20.8)	31 (15.6)
2–5 years	78	12 (50.0)	66 (33.2)
6–10 years	49	5 (20.8)	44 (22.1)
11–15 years	27	2 (8.3)	25 (12.6)
16–20 years	7	0 (0)	7 (3.5)
20+ years	26	0 (0)	26 (13.1)

**Table 2:** Participant baseline clinical information.

	< 18 years N (column %) Total = 24	≥18 years N (column %) Total = 201	P value
**Pancreatitis diagnosis**			
Acute pancreatitis	1 (4.2)	11 (5.5)	1
recurrent acute pancreatitis	9 (37.5)	19 (9.5)	<0.001
Auto immune pancreatitis	0 (0)	4 (2.0)	1
Chronic pancreatitis	4 (16.7)	134 (66.7)	<0.001
Hereditary pancreatitis	8 (33.3)	16 (8.0)	<0.001
Other	2 (8.3)	17 (8.5)	1
**Type of genetic mutation**			
PRSS1	7 (29.2)	12 (6.0)	<0.001
SPINK	6 (25.0)	6 (3.0)	<0.001
CFTR	5 (20.9)	13 (6.5)	0.014
CTRC	0 (0)	0 (0)	N/A
Other	0 (0)	3 (1.5)	1
Unknown/ Missing Data	6	172	
**Diabetes mellitus**			
Yes	2 (8.3)	53 (26.3)	0.047
No	22 (91.7)	140 (69.7)	
Unknown/ Missing Data	0	8	
**Type of DM**			
Type 1 DM	0 (0)	7 (13.2)	0.628
Type 2 DM	0 (0)	19 (35.9)	
Type 3c DM	2 (100)	24 (45.3)	
Unknown/ Missing Data	0	3	
**Exocrine Pancreatic insufficiency**			
Yes	10 (41.7)	100 (49.8)	0.374
No	11 (45.8)	73 (36.3)	
Unknown/ Missing Data	3	28	

There was no significant difference in exocrine pancreatic insufficiency or DM between pediatric and adults (Table 2).

### Symptoms and diagnoses known to be associated with pancreatitis in adults compared to children- secondary outcomes

**Fig 3:**
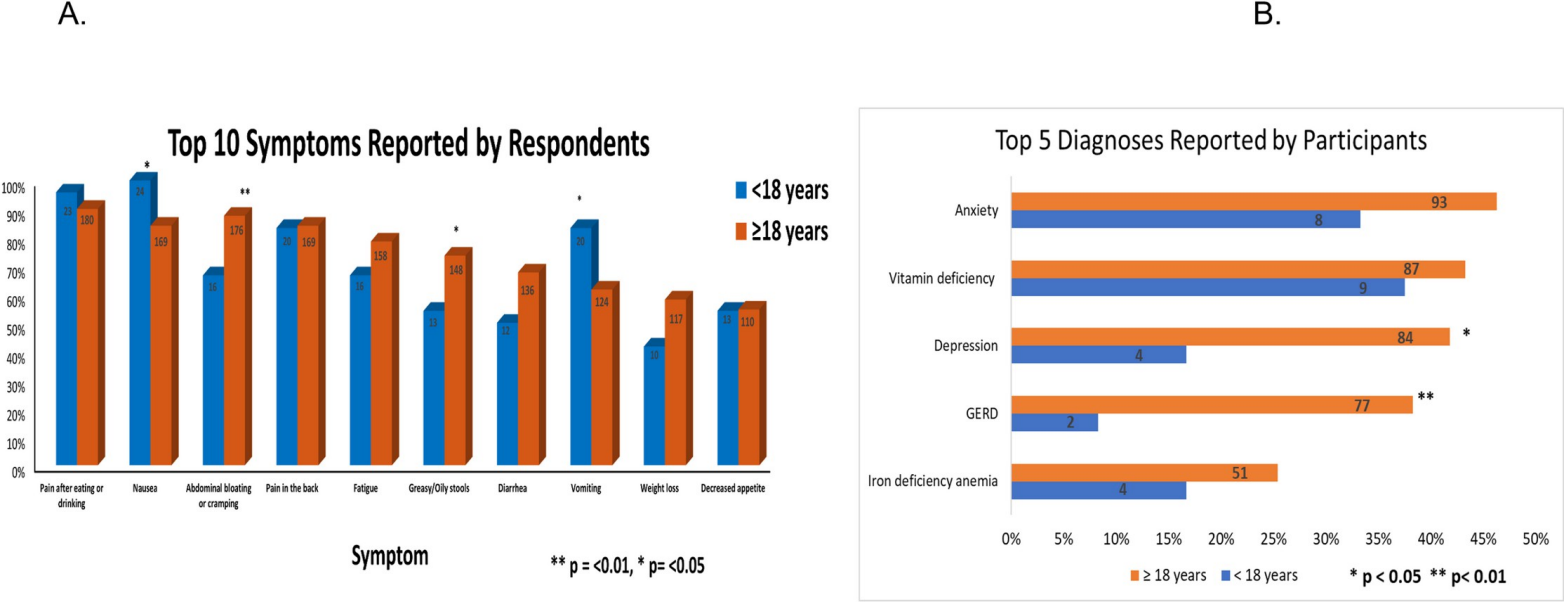
A: Bar chart with top 10 symptoms reported by survey participants. B: Bar chart with top 5 diagnoses reported by survey participants.

Table 3 shows the symptoms commonly associated with pancreatitis that were reported by the pediatric and adult cohorts. When comparing the pediatric cohort to the adult cohort, the following symptoms were significantly different between the two cohorts: abdominal bloating or cramping (16 (66.7%) vs. 176 (87.6%) p = 0.006), nausea (24 (100%) vs. 169 (84.1%) p = 0.030), vomiting (20 (83.3%) vs. 124 (61.7%) p = 0.043) and greasy or oily stools (13 (54.2%) vs. 148 (73.6%) p = 0.046) (Fig 3A).

**Table 3:** Participant reported symptoms commonly reported in pancreatitis.

	Overall Total = 225	< 18 years N (%) Total = 24	≥18 years N (%) Total = 201	*P value
Pain after eating or drinking	200 (88.9)	20 (83.3)	180 (90.0)	0.318
Nausea	193 (85.8)	24 (100.0)	169 (84.1)	0.030
Abdominal bloating or cramping	192 (85.3)	16 (66.7)	176 (87.6)	0.006
Pain in your back	189 (84.0)	20 (83.3)	169 (84.1)	1
Fatigue or feeling tired frequently	174 (77.3)	16 (66.7)	158 (78.6)	0.187
Greasy/Oily stools	161 (71.6)	13 (54.2)	148 (73.6)	0.046
Diarrhea or loose stools	148 (65.8)	12 (50.0)	136 (67.7)	0.085
Vomiting	144 (64.0)	20 (83.3)	124 (61.7)	0.043
Weight loss	127 (56.4)	10 (41.7)	117 (58.2)	0.122
Decreased appetite	123 (54.7)	13 (54.2)	110 (54.7)	0.959
Constipation/ hard stools	116 (51.6)	14 (58.3)	102 (50.8)	0.482
Frequent Dehydration	100 (44.4)	11 (45.8)	89 (44.3)	0.885
Poor weight gain	77 (34.2)	10 (41.7)	67 (33.3)	0.416
Have you experienced any of these symptoms related to pancreatitis? (choice = Other)	24 (10.7)	2 (8.3)	22 (11.0)	1
Patients with pancreatitis often report other symptoms that have not been well-documented. Have you experienced any of the symptoms noted below? (choice = Other)	11 (4.9)	2 (8.3)	9 (4.5)	0.332
Patients with pancreatitis often report other symptoms that have not been well-documented. Have you experienced any of the symptoms noted below? (choice = None of these apply to me)	3 (1.3)	0 (0)	3 (1.5)	1
Have you experienced any of these symptoms related to pancreatitis? (choice = None of the above symptoms apply to me)	0 (0)	0 (0)	0 (0)	N/A

*p value compares symptoms in children (< 18 years) to adults ≥ 18 years old

### Symptoms and diagnoses that are rarely reported in association with pancreatitis—extra pancreatic manifestations- primary outcomes

The top 5 extra pancreatic symptoms were reported in > 40% of the cohort and included feeling cold in 105 (46.7%), joint pains in 103 (45.8%), dizziness in 98 (43.6%), headache 95 (42.2%), night sweats 93 (41.3%) (Table 4). Interestingly, when comparing these symptoms between pediatrics and adults, feeling cold (4 (16.7%) vs. 101 (50.3%), and night sweats (3 (12.5%) vs. 90 (44.8%) were significantly different (p values = 0.002).

**Table 4:** Participant extra pancreatic symptoms.

	Overall Total = 225	< 18 years N (%) Total = 24	≥18 years N (%) Total = 201	P value
Feeling cold	105 (46.7)	4 (16.7)	101 (50.3)	0.002
Joint pains	103 (45.8)	9 (37.5)	94 (46.8)	0.389
Dizziness	98 (43.6)	9 (37.5)	89 (44.3)	0.527
Headache	95 (42.2)	10 (41.7)	85 (42.3)	0.954
Waking up sweating at night	93 (41.3)	3 (12.5)	90 (44.8)	0.002
Rapid Pulse	86 (38.2)	9 (37.5)	77 (38.3)	0.939
Dry eyes	78 (34.7)	4 (16.7)	74 (36.8)	0.068
Brittle nails	73 (32.4)	6 (25.0)	67 (33.3)	0.409
Brittle Hair	71 (31.6)	5 (20.8)	66 (32.8)	0.352
Hair loss	66 (29.3)	3 (12.5)	63 (31.3)	0.060
Sinus issues or chronic rhinosinusitis	59 (26.2)	5 (20.8)	54 (26.9)	0.525
Extreme cold or pain in the fingers or toes after exposure to cold	58 (25.8)	3 (12.5)	55 (29.4)	0.142
Excessive Sweating during the Day	56 (24.9)	7 (29.2)	49 (24.4)	0.608
Low body temperature/ Hypothermia	50 (22.2)	4 (16.7)	46 (22.9)	0.609
Mouth sores	44 (19.6)	5 (20.8)	39 (19.4)	0.791
Fever	44 (19.6)	7 (29.2)	37 (18.4)	0.209
Abdominal pancreas pain episode flared by menstrual cycle	44 (19.6)	8 (33.3)	36 (17.9)	0.072
Fragile or Weak Bones	42 (18.7)	3 (12.5)	39 (19.4)	0.581
Vertigo	40 (17.8)	3 (12.5)	37 (18.4)	0.474
Rash	37 (16.4)	5 (20.8)	32 (15.9)	0.561
Whitish or Purplish discoloration of fingers or toes	33 (14.7)	4 (16.7)	29 (14.4)	0.761
Joint swelling	32 (14.2)	3 (12.5)	29 (14.4)	1
Swollen lymph nodes or glands	31 (13.8)	3 (12.5)	28 (13.9)	0.848
Gastrointestinal bleed such as melena	22 (9.8)	0 (0)	22 (11.0)	0.141
Swollen Tongue	9 (4.0)	1 (4.2)	8 (4.0)	0.965

*p value compares symptoms in children (< 18 years) to adults ≥ 18 years old

The top 5 overall diagnoses reported by the cohort were anxiety 101 (44.9%), vitamin deficiencies 96 (42.7%), depression 88 (39.1%), gastroesophageal reflux disease 79 (35.1%), and iron deficiency anemia 55 (24.4%) (Table 5 and Fig 3B). Depression and gastroesophageal reflux disease were significantly reported less by the pediatric cohort with p values of 0.025 and 0.003 respectively. Furthermore, 6(25%) of the pediatric cohort reported no extra-pancreatic diagnoses compared to only 16 (8%) in adults (p = 0.018).

**Table 5:** Participant reported diagnoses.

	Overall Total = 225	< 18 years N (%) Total = 24	≥18 years N (%) Total = 201	*P value
Anxiety	101 (44.9)	8 (33.3)	93 (46.3)	0.280
Vitamin Deficiency	96 (42.7)	9 (37.5)	87 (43.3)	0.588
Depression	88 (39.1)	4 (16.7)	84 (41.8)	0.025
Gastroesophageal reflux disease	79 (35.1)	2 (8.3)	77 (38.3)	0.003
Iron deficiency anemia	55 (24.4)	4 (16.7)	51 (25.4)	0.455
Other	26 (11.6)	1 (4.2)	25 (12.4)	0.325
Abdominal adhesions	25 (11.1)	0 (0)	25 (12.4)	0.085
Other Autoimmune Conditions	25 (11.1)	0 (0)	25 (12.4)	0.085
None	22 (9.8)	6 (25.0)	16 (8.0)	0.018
Hypothyroidism	21 (9.3)	0 (0)	21 (10.4)	0.089
Functional gastrointestinal disorder	16 (7.11)	2 (8.3)	14 (7.0)	0.682
Kidney disease	15 (6.7)	2 (8.3)	13 (6.5)	0.666
Eczema	11 (4.9)	2 (8.3)	9 (4.5)	0.332
Raynaud’s syndrome	9 (4.0)	1 (4.2)	8 (4.0)	1
Ulcerative colitis	9 (4.0)	0 (0)	9 (4.5)	0.602
Hyperthyroidism	8 (3.6)	1 (4.2)	7 (3.5)	0.601
Peptic ulcer disease	8 (3.6)	0 (0)	8 (4.0)	1
Sjogren’s syndrome	7 (3.1)	0 (0)	7 (3.5)	1
Celiac disease	7 (3.1)	1 (4.2)	6 (3.0)	0.551
Crohn’s disease	7 (3.1)	0 (0)	7 (3.5)	1
Cystic Fibrosis	7 (3.1)	2 (8.3)	5 (2.5)	0.164
Rheumatoid arthritis	6 (2.7)	0 (0)	6 (3.0)	0.372
Lupus	4 (1.8)	0 (0)	4 (2.0)	1
(POTS) syndrome	4 (1.8)	0 (0)	4 (1.9)	1
Hypermobility syndrome/ Ehlers Danlos syndrome	3 (1.3)	1 (4.2)	2 (1.0)	0.288
Psoriasis	2 (0.8)	0 (0)	2 (1.0)	1
Panniculitis	1 (0.4)	0 (0)	1 (0.5)	1
Vitiligo	0 (0)	0 (0)	0 (0)	N/A

*p value compares diagnoses in children (< 18 years) to adults ≥ 18 years old

## Discussion

In this study we asked adults and children with pancreatitis about their experience with extra pancreatic symptoms and diagnosis. Almost all participants reported at least one extra pancreatic symptom (that is a symptom not commonly reported in pancreatitis) signifying the burden of the disease. The extra-pancreatic symptoms and diagnoses are present in most patients with pancreatitis and are more likely to occur in adults with pancreatitis than children. To our knowledge, this is the first study to incorporate a patient reported perspective via an anonymously administered survey to determine the prevalence of extra-pancreatic manifestations in a cohort of patients with pancreatitis.

Other organ-system involvement have been described with other diseases such as inflammatory bowel disease [16] and may parallel or precede intestinal activity. Reports in pancreatitis have been limited to case reports, case series, single center studies and a few systematic reviews [17–20]. A systematic review by Brown et al. revealed that a third of patients with acute pancreatitis will develop infectious complications with respiratory infection and bacteremia being the most common [17]. Another study by Kothari et al. [18] revealed an incidence of 34% of extra-pancreatic complications in adults admitted to the ICU with AP. These complications were associated with increased length of stay and the non-infectious complications increased mortality [18].

In our cohort, 90% of participants reported at least one extra-pancreatic symptom or diagnosis. This contrasts with the retrospective study by Abbasi et al. [21] where ∼ 20% of patients (AA and Hispanic) in their cohort had extra-pancreatic manifestations. In that study, gastrointestinal (GI) bleed was the most common extra-pancreatic manifestation in about 22% of their patients. However, fatigue was the most common extra-pancreatic symptom in cohort and was reported by 79% of adults and 67% of children [21]. In this study, GI bleed was reported by about 11% of adults and no child reported a GI bleed. It is possible that the differences in symptom prevalence observed between our study and the retrospective study by Abbasi and colleagues may be due to the cohort differences in demographics (AA and Hispanic versus Caucasian) and diagnosis of pancreatitis (acute pancreatitis versus chronic pancreatitis) or even a reporting bias.

Surprisingly, many participants reported symptoms associated with certain diseases surveyed, however, the percentage of reported diagnoses of these diseases were lower than the percentage of respondents reporting the symptoms associated with the diseases. This could represent an under-reporting of symptoms by these patients to their providers to necessitate further workup and proper diagnosis or missed opportunity by providers to make the diagnosis. We hope that these findings will prompt further studies in this area to determine the true prevalence of these diagnoses.

Although our study reveals important findings on the commonly reported symptoms in pancreatitis and extra pancreatic manifestations in a pancreatitis cohort, limitations still exist. Some of the limitations are in the sample size, the possible recall bias, possibility of gender bias as most subscribers to Mission: Cure mailing list identify as females and the lack of objective diagnosis by medical provider as a confirmation of those symptoms. Additionally, it is possible that symptoms reported may be related to other diagnoses not reported by these participants. However, the strengths of our study are in the inclusion of adult and children in the cohort and focusing on the important aspects from the patient’s point of view, as these may have been missed if this survey and study was driven by medical providers only. Lastly, the wide representation of a random international sample is an additional strength to the study that would allow for generalizability.

## Conclusion

Patients with pancreatitis report extra-pancreatic symptoms with limited extra-pancreatic diagnoses. Improved awareness of these symptoms with subsequent diagnoses and treatment may reduce the frequency, severity of complications of pancreatitis and most importantly improve outcomes that are most impacting the patients with pancreatitis.

## Supporting information

S1 Checklist STROBE statement—checklist of items that should be included in reports of observational studies. (DOCX)
